# Maraviroc for Stroke Recovery (MASTER): protocol for a phase 2 double-blind placebo-controlled randomised clinical trial

**DOI:** 10.1136/bmjopen-2025-109554

**Published:** 2026-05-03

**Authors:** Nicolas Broc, Gabriel Byczynski, Elisabeth Dirren, Emmanuel Carrera

**Affiliations:** 1 Geneva University Hospital, Geneva, Switzerland; 2 Faculty of Medicine, University of Geneva, Geneva, Switzerland

**Keywords:** Clinical Trial, Randomized Controlled Trial, Magnetic resonance imaging, Stroke, REHABILITATION MEDICINE

## Abstract

**Introduction:**

Despite advances in acute treatment, stroke remains the first cause of acquired disability. Today, there is no effective pharmacological therapy to improve recovery beyond the acute phase. Preclinical studies suggest that inhibition of the C-C chemokine Receptor 5 (CCR5) may promote recovery by enhancing plasticity in the peri-infarct cortex. However, the role of CCR5 to improve outcome after ischaemic stroke in humans is unknown.

**Methods and analysis:**

MAraviroc for STrokE Recovery is a phase II, single-centre, randomised, double-blind, placebo-controlled trial. The aim is to assess the efficacy and safety of the CCR5 antagonist Maraviroc for improving motor recovery of the upper limb after ischaemic stroke. 80 ischaemic stroke patients with moderate but incomplete upper extremity motor impairment will be enrolled within 7 days of onset. Participants will be randomised (1:1) to receive either oral maraviroc (300 mg two times per day) or placebo for 90 days in addition to standard rehabilitation therapy. The primary outcome is upper limb motor function assessed using the Fugl-Meyer Assessment for the Upper Extremity at day 90. Secondary outcomes include motor learning skills and plasticity in the peri-infarct region assessed using MRI connectivity and spectroscopy at 90 days.

**Ethics and dissemination:**

The study protocol has been reviewed and approved by the Geneva Competent Ethics Committee (Commission Cantonale d’Éthique, CEC; Reference Number: CCER 2024-02359) and Swissmedic (Swiss Agency for Therapeutic Products). Written informed consent will be obtained from all participants. Study results will be disseminated through peer-reviewed publications and scientific conferences.

**Trial registration number:**

2024-02359; NCT07080567.

**STRENGTHS AND LIMITATIONS OF THIS STUDY**

- First study to initiate treatment in the acute phase of stroke (≤7 days after onset) to evaluate the efficacy of maraviroc to improve motor function.
- Use of standardised clinical outcomes combined with MRI-based measures to determine the clinical and neural correlates of drug effect.
- This study is limited to evaluating the effect of maraviroc on upper extremity function.
- The study assesses only a single treatment regimen.

## Introduction and rationale

 Persistent neurological deficits are frequent after stroke. Today, there are no effective pharmacologic therapies to improve stroke recovery beyond the acute phase.^1^ Pharmacological interventions offer advantages: they are not reliant on patient motivation, easy to administer and require fewer medical resources.

Maraviroc is a small-molecule inhibitor of the CCR5 receptor (C-C chemokine Receptor 5).^2^ This treatment has been developed for HIV therapy following the discovery that CCR5 is an essential co-receptor for the entry of the virus into host cells.^3^ This strategy was further supported by the observation that individuals homozygous for the CCR5-Δ32 mutation, which prevents surface expression of the receptor, were naturally resistant to HIV infection.^3^

CCR5 inhibition has emerged as a potential strategy to enhance plasticity after stroke in rodent models. Nevertheless, the timing of CCR5 inhibition appears critical, as transient poststroke pharmacological inhibition improves plasticity but constitutional genetic deletion may worsen outcomes.^4^

In rodents, CCR5 inhibition using maraviroc was associated with increased dendritic spine density in peri-infarct cortex and increased projecting axons between the peri-infarct cortex to the homologous contralateral cortex.^5^ These plastic changes correlated with behavioural performance, even beyond the acute phase.^5^ However, findings from genetic models complicate the interpretation of the role of CCR5 in recovery. Following middle cerebral artery occlusion, infarct size was markedly increased in constitutionally CCR5-deficient mice.^6^ Ubiquitous lack of CCR5 expression in mice appears deleterious for stroke recovery, on the contrary to the benefit of pharmacological inhibition by maraviroc.

In humans,^2 7^ no data have yet confirmed the potential of maraviroc after stroke. A loss-of-function CCR5 mutation was associated with a better recovery in ischaemic stroke patients.^5^ Pharmacological inhibition of the CCR5 receptor appears as a promising strategy to improve recovery after stroke.

Here, we present MAraviroc for STrokE Recovery (MASTER) trial protocol, designed to investigate the efficacy and mechanisms of action of maraviroc to improve motor recovery after ischaemic stroke.

## Methods

### Hypothesis

Maraviroc improves motor recovery after ischaemic stroke compared with placebo.

### Study design

MASTER is a single-centre, patient and investigator-blinded, randomised, placebo-controlled, phase 2 clinical trial. It assigns patients with acute ischaemic stroke (within 7 days of onset) and motor impairment to either maraviroc (2×300 mg/day) or placebo for 90 days. The trial will randomise at a 1:1 ratio, powered to assess the superiority of maraviroc over placebo. The trial will be conducted at the Stroke Centre of the Geneva University Hospital, Switzerland.

### Recruitment

All patients admitted to the Stroke Centre will be screened during the daily clinical rounds conducted by board-certified stroke neurologists to ensure unbiased recruitment. Eligible patients will be enrolled within 7 days of onset.

### Eligibility criteria

The inclusion and exclusion criteria for the MASTER trial are shown in box 1.

Box 1 **Inclusion and exclusion criteria of efficacy and safety of the MASTER trial**

**Inclusion criteria**

1. Informed consent by signature.
2. Older than 18 years old.
3. Acute ischaemic stroke (stroke onset within 7 days of randomisation).
4. Contralateral, unilateral, incomplete upper limb paresis.
  - Unilateral motor impairment with a Fugl-Meyer Upper-Extremity Score of less than 63/66.
  - Residual voluntary finger extension of at least 10°.

**Exclusion criteria**

1. Pregnancy or lactation or positive pregnancy test in women of childbearing age (due to contraindication of maraviroc).
2. Prestroke handicap with modified Rankin Scale >2.
3. Diseases affecting motor function (eg, Parkinson’s disease, amyotrophic lateral sclerosis, amputation).
4. Participation in another study with an investigational medicinal product within 30 days of randomisation.
5. Enrolment of the investigator, his/her family members, employees and other dependent persons.
6. Known hypersensitivity or allergy to maraviroc, peanuts, soy and/or mannitol.
7. History of significant liver disease, hepatitis, elevated liver function tests (greater than 1.5 times the upper limit of normal).
8. History of significant renal disease or end stage renal disease/dialysis, acute renal injury, creatinine clearance of less than 30 mL/min/1.73 m^2^.
9. Patients with cardiovascular comorbidities and risk for orthostatic hypotension (eg, symptomatic heart failure, active coronary disease).
10. HIV infection.
11. Concomitant use of strong CYP3A4 inhibitors.
12. Concomitant use of strong CYP3A4 inducers.

MASTER, Maraviroc for Stroke Recovery.

### Blinding and randomisation

Trial participants, care providers, outcome assessors, investigators and the study team will be blinded to treatment allocation throughout the trial. Blinding is maintained by the pharmacy of the Clinical Trial Unit (CTU), which prepares identical capsules containing either maraviroc 150 mg or placebo, indistinguishable in appearance and packaging. Each treatment bottle is labelled with a unique randomisation number. All trial data are managed through the SecuTrial clinical database, which is fully blinded to treatment assignment. Study unblinding will occur only after database lock and validation of the final statistical analysis plan by the investigators and sponsor. Unblinding of an individual participant’s allocation is only permitted in the event of a serious adverse event (SAE), when knowledge of the treatment arm is essential for clinical decision-making.

Randomisation (1:1) will be conducted by an independent statistician from the CTU at the Geneva University Hospital. A computer-generated list will be created using random permuted blocks of variable sizes (2, 4 and 6) to ensure allocation concealment. Stratification will be performed by:

- Side of infarct (left vs right hemisphere)
- Degree of finger extension (Fugl-Meyer Extension Subscore: 1 or 2 points)

This results in four randomisation strata, ensuring balance across treatment arms with respect to the laterality and severity of motor impairment.

### Intervention

The experimental intervention will be the administration of Maraviroc. The dosage will be 300 mg two times per day orally (2 capsules of 150 mg in the morning and 2 capsules of 150 mg in the evening). The experimental product will be reconditioned by the HUG pharmacy (crushed, mixed with mannitol and prepared in a capsule) from the original product to match the placebo. Placebo will be mannitol in identical capsules.

All patients will be treated with standard of care including secondary stroke prevention strategies in line with institutional and European guidelines (European Stroke Organisation guidelines on secondary stroke prevention^8^). All participants will receive standard neurorehabilitation according to our institutional stroke rehabilitation programme, aligned with European Stroke Organisation guidelines,^9^ which is currently recommended to include a minimum of 20 hours of intensive repetitive arm training 3–5 times per week over 4–6 weeks. During inpatient rehabilitation, detailed information on therapy content and focus (including upper-limb-specific and task-oriented training) will be recorded. During outpatient rehabilitation, rehabilitation exposure will be quantified by weekly therapy hours. This will enable controlling both for the number of hours of therapy received and percentage of patients meeting ESO guideline requirements, based on the reported therapy content.

### Criteria for discontinuing or modifying allocated interventions

There will be no dose modifications in this study, as patients whose dose should be reduced (presenting with estimated glomerular filtration rate (eGFR) <80 mL/min or prescribed concomitant strong CYP3A4 inhibitors) are excluded by design. Allocated intervention will be discontinued in the following situations:

- Pregnancy.
- Liver toxicity: liver function tests (LFTs) reach Common Terminology Criteria for Adverse Events (CTCAE) grade ≥3 with bilirubin ≥1.5× the upper limit of normal and normal alkaline phosphatase, or LFTs reach grade ≥3 alone.
- Renal impairment: eGFR<30 mL/min.
- Cardiovascular: significant symptoms or findings of heart failure (eg, severe hypotension, arrhythmia) as assessed through ECG, vitals and clinical evaluation, treatment may be stopped based on clinical judgement.
- Infections or hypersensitivity: serious infection, significant eosinophilia or hypersensitivity reactions.
- Other safety concerns: Any emerging AE deemed to pose undue risk to the participant will lead to discontinuation, as determined by the investigator.

All safety-related discontinuations will be documented and reviewed by the study team.

### Relevant concomitant care permitted or prohibited during the trial

The use of strong CYP3A4 inhibitors or inducers is prohibited and constitutes an exclusion criterion. All other pharmacological treatments are permitted and will be recorded. Non-pharmacological supportive therapies, such as physiotherapy, occupational therapy and speech therapy, are allowed and encouraged as part of standard post-stroke care and will continue throughout the study.

### Strategies to improve adherence to interventions

Participants will receive clear verbal and written instructions regarding the study intervention schedule. Study medication will be dispensed at designated visits, and participants will be asked to return all unused tablets and packaging at subsequent visits (visits 3, 4 and 5). Adherence will be assessed by tablet counts and direct questioning. Tablets not returned will be assumed to have been taken.

Phone follow-up calls will be conducted between visits (weeks 2, 6 and 10) to monitor adherence, reinforce compliance and identify potential AEs. Compliance will be defined as taking 80%–120% of the prescribed doses. Non-compliance will be documented and reviewed. A full dispensing log will track all medication dispensed, returned and destroyed.

### Data collection and management

**Figure 1:**
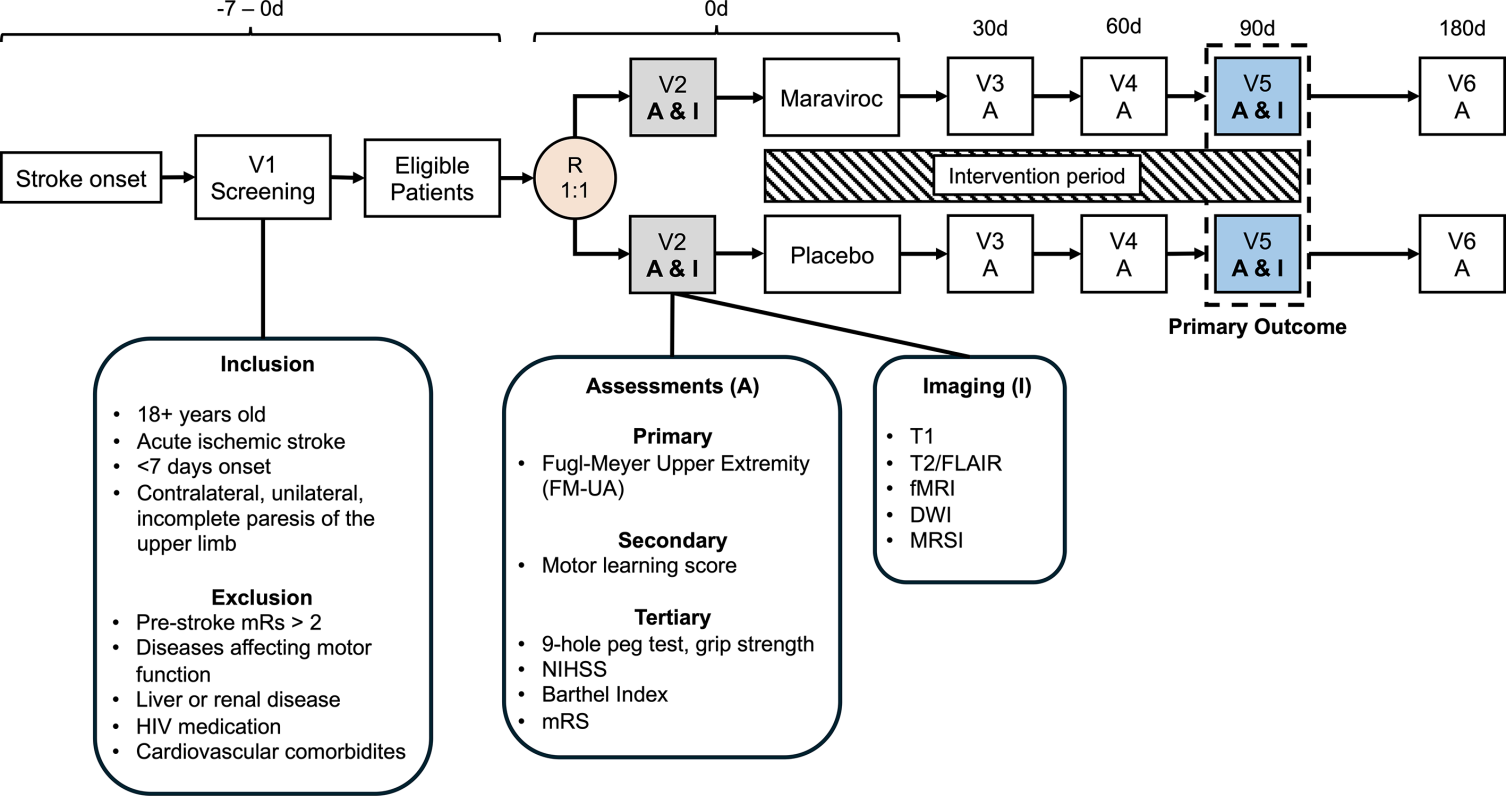
Flow chart of the MASTER trial. V1 screening includes prescreening of patients for inclusion criteria and inclusion for additional screening of exclusion criteria. Randomisation occurs within 7 days of stroke onset. V2 includes randomisation and initial assessments (imaging and clinical), carried out within 24 hours of beginning of intervention medication. V3 and V4 include one clinical assessment. V5 is the primary outcome time point, including both imaging and clinical assessments. V6 represents the 180 days follow-up. Telephone check-ins occur between each in-person visit. MASTER, Maraviroc for Stroke Recovery; mRS, modified Rankin Scale; NIHSS, National Institutes of Health Stroke Scale; FLAIR, Fluid-Attenuated Inversion Recovery; DWI, Diffusion-Weighted Imaging; MRSI, Magnetic Resonance Spectroscopic Imaging.

All outcomes will be assessed using standardised procedures by trained personnel. Data will be collected at baseline and at 30, 60, 90 and 180 days poststroke. Participants who discontinue the intervention will be encouraged to complete all assessments. When in-person visits are not feasible, alternative follow-up (eg, home or phone visits) may be arranged. The patient timeline is shown in figure 1.

### Primary outcome

#### Upper Extremity Fugl-Meyer Assessment score at 90 days poststroke

The Upper Extremity Fugl-Meyer Assessment (FMA-UE) will be conducted at all time points by trained occupational therapists or board-certified neurologists. This 0–66 point scale is widely used in stroke research for its reliability and sensitivity to change.^9^ The FMA-UE is a validated, reliable tool for assessing upper limb motor function in stroke patients.^9^ The minimal clinically important difference (MCID) is 12.4 points for patients with subacute, moderately severe stroke.^10^ All raters will undergo training before study initiation.

### Secondary outcomes

Motor learning score (ability of a subject to learn a finger tapping sequence task with the paretic hand) at 90 days poststroke.

The motor sequence learning task assesses procedural motor learning capacity, relevant to rehabilitation outcomes and has been validated in previous studies within our own group.^11^ It will be administered at baseline and at 90 days. It consists of a finger tapping task using a commercial five-button response box. Data will be collected digitally and stored securely for analysis.

### Other outcomes

Hand dexterity (9 Hole Peg Test), grip and pinch strength (Hand/finger dynamometer), both sensitive and reliable measures of upper limb recovery,^12^ and clinical measures including National Institutes of Health Stroke Scale, modified Rankin Scale and Barthel Index. They are performed by trained, certified staff using validated tools at each visit.

Changes in functional connectivity between peri-infarct regions and motor network node, specifically focusing on the changes in structural connectivity (eg, fractional anisotropy) between peri-infarct regions and motor network nodes, changes in microstructural integrity within the peri-infarct area (eg, neurite density), and changes in neuronal viability in the peri-infarct area between randomisation and the 90-day follow-up (eg, concentrations of *N*-acetylaspartate, Creatinine, Glutamate-Glutamine complex).^13^ They will be assessed via MRI (resting-state functional MRI, multi-shell Diffusion-Weighted Imaging, 3-dimensional MR spectroscopy), using a standardised protocol on a 3T scanner. MRI data will be processed by blinded staff.

Laboratory tests include baseline blood analyses, genotyping for the CCR5-Δ32 mutation and biobanking for further biomarker studies (baseline, 90 days).

### Safety outcomes

CTCAE v5.0-defined AEs through 180 days, including major adverse cardiovascular events (ie, acute myocardial infarction, acute coronary syndrome, stroke, heart failure, cardiovascular and all-cause mortality, revascularisation), liver-related AEs and infectious complications. SAEs will be tracked continuously through 180 days post randomisation.

Blood samples will be collected at each visit for safety monitoring. Participants of childbearing potential will undergo a urine pregnancy test at each visit. Samples will be processed and stored under standardised conditions.

### Statistical methods

#### Sample size estimate

We estimated a sample size of 80 participants, based on a MCID for FMA-UE of 12.4 points in the early subacute phase of stroke recovery,^10^ a pooled SD of 17.5, based on the FLAME trial,^14^ a 5% type I error and 80% power and accounting for a 20% dropout or exclusion rate. We used the results of the FLAME randomised clinical trial (RCT), which investigated the effect of fluoxetine on stroke recovery. We based our estimation on this study because it is a large pharmacological RCT focusing on a similar population including patients with moderately severe motor deficits early after ischaemic stroke. No studies investing maraviroc after stroke currently exist.

### Statistical methods for primary and secondary outcomes

Two analysis sets will be used: an intention-to-treat population (all randomised participants who received ≥1 dose, analysed per allocation) and a per-protocol population (excluding those with major protocol deviations, eg, <80% medication adherence). Missing data will not be imputed.

The primary outcome (FMA-UE) will be compared between groups using a t-test or non-parametric equivalent. An adjusted analysis using multivariate linear regression will account for stratification variables (infarct side, finger extension ability) as well as other variables like CCR5-Δ32 loss-of-function mutation carrier status to account for confounding effects on motor recovery and treatment response. Secondary outcomes will be analysed via regression models adjusted for the same covariates. Linear mixed models will include time point and group as fixed effects and participant ID as a random effect to account for repeated measures. To assess whether maraviroc promotes recovery via peri-infarct plasticity, causal mediation analysis will estimate direct and indirect effects (via changes in connectivity/metabolism from baseline to 90 days). The analysis includes three regression steps, with the indirect effect tested via the Sobel method. Weekly rehabilitation hours (during the first 90 days) will be included as a covariate. A similar approach will be applied to motor learning outcomes. Safety analyses will summarise the number and proportion of participants with ≥1 SAE, AE of special interest, or death by day 90 in each group.

Analyses will be conducted in R, MATLAB or Python using appropriate packages. Imaging data will be processed with FSL, FreeSurfer, SPM and DSI Studio, depending on modality.

### Trial coordination and data safety monitoring

The coordinating centre for this trial is based at HUG, where the sponsor-investigator, trial management team and all operational units are located.

Day-to-day trial operations are managed by a dedicated research team, including coinvestigators and study coordinators. This team oversees patient recruitment, protocol adherence, scheduling of assessments and communication among the trial’s various operational components. The research team will meet regularly—on a weekly or biweekly basis—to ensure smooth trial progress and resolve any logistical or clinical issues.

The CTU at HUG (Geneva University Hospital) will provide monitoring services via a dedicated monitoring plan that has been approved by the ethics committee and regulatory authorities. Statistical support will be ensured by the Department of Clinical Epidemiology, and biological and imaging analyses will be conducted by specialised laboratory services at HUG. These groups will collaborate with the coordinating centre to maintain data quality and regulatory compliance throughout the trial.

A Data Safety Monitoring Board (DSMB) will be established to independently monitor participant safety and oversee safety-related aspects of the study. The DSMB will remain blind to treatment allocation and will make decisions based on expected incidence of AEs in the stroke population. Decision to suspend, terminate or amend the trial belongs to the sponsor-investigator. All AEs and SAEs will be collected, fully investigated, and documented from the time of informed consent until the completion of the last protocol-specific procedure, including the 6-month follow-up (Day 180±14). There are no planned interim analyses for efficacy. The Geneva University Hospital has contracted an insurance to compensate patients who suffer harm from trial participation.

### Patient and public involvement

Patients or the public were not involved in the design, conduct, reporting or dissemination plans of our research

### Study current stage

Recruitment started on 14 July 2025 and is to be completed by December 2027.

## Discussion

The MASTER trial represents an innovative pharmacological strategy to improve motor function after ischaemic stroke. If maraviroc demonstrates efficacy to improve motor function, while maintaining an acceptable safety profile, it could be of major clinical relevance in the management of stroke patients. This study could also clarify the role of pharmacological approaches in poststroke motor rehabilitation.

Taking advantage of the organisation of the Stroke Centre of our institution, we will be able to enrol patients within the first week after stroke onset. This early inclusion allows us to provide novel insights into the effects of initiating drug therapy in the acute to subacute phase, in contrast to most other studies that have included patients at later time points. The originality of MASTER is its multimodal design. The trial integrates measures of motor function and motor learning but also measures of functional and structural plasticity using high-resolution MRI. This comprehensive approach enables the evaluation of clinical impact of maraviroc and explores the underlying mechanisms.

The methodological strengths of the study include stratification by infarct side and motor severity, blinding procedures and active monitoring by the local CTU. In addition, the inclusion of biomarker analysis and genotyping aims to identify which patients are most likely to benefit from the intervention, paving the way for more personalised therapeutic strategies.

## Ethics statements

### Ethics approval

The study protocol has been reviewed and approved by the Geneva Competent Ethics Committee (Commission Cantonale d’Éthique, CEC; Reference Number: CCER 2024-02359) and Swissmedic (Swiss Agency for Therapeutic Products). Approval was granted in accordance with the principles set forth in the current Declaration of Helsinki, the International Council for Harmonisation Good Clinical Practice guidelines, Swiss law, and the Ordinance on Clinical Trials (ClinO).

Prior to any study-related procedures, written informed consent is obtained from all participants by a study investigator (see online supplemental material 1 for an example of the participant informed consent form). In cases where participants lack the capacity to provide consent, their legally authorised representatives provide consent on their behalf, in compliance with applicable regulations. Specific consent for further use of participant data and biological specimens will be obtained.

## Supplementary material

10.1136/bmjopen-2025-109554 online supplemental file 1
