# Supplementary material for: Maraviroc for Stroke Recovery (MASTER): protocol for a phase 2 double-blind placebo-controlled randomised clinical trial
Source: BMJ Open. 2026 May 3;16(4):e109554. doi: 10.1136/bmjopen-2025-109554 (PMC13141164; doi:10.1136/bmjopen-2025-109554)
Supplement: online supplemental file 1 [file bmjopen-16-4-s001.pdf]

Hôpitaux
Universitaires
Genève

Demande de participation à une recherche médicale

Titre de l'étude : Étude de phase II sur l'efficacité sur la récupération motrice et la sécurité du maraviroc par rapport au placebo après un accident vasculaire cérébral.

Titre simplifié : MASTER.

Madame, Monsieur,

Nous aimerions vous présenter notre étude clinique et vous inviter à y participer. Avant qu'un nouveau médicament puisse être prescrit, des recherches sont en effet nécessaires pour savoir comment ce médicament agit.

En médecine, une telle recherche s'appelle une **étude clinique**. Dans cette étude, nous voulons découvrir quel effet le maraviroc a sur la récupération motrice chez les personnes qui, comme vous, ont souffert d'un accident vasculaire cérébral (AVC) ischémique et qui sont hospitalisés dans notre service. C'est pourquoi nous vous demandons si vous souhaitez participer à cette étude.

Votre participation est volontaire. Ce **formulaire d'information** doit vous aider à prendre votre décision. Vous pouvez poser toutes vos questions lors d'un **entretien avec l'un des médecins investigateurs**. Cette personne est responsable du suivi des participant.e.s de l'étude. Si vous souhaitez participer, veuillez signer la **déclaration de consentement** à la fin du document. Par votre signature, vous attestez avoir lu et compris les informations fournies. Si vous ne comprenez pas quelque chose, n'hésitez pas à demander des précisions à **l'un des médecins investigateurs**.

Le formulaire d'information et de consentement se compose de quatre parties :

Partie 1 L'essentiel en bref
Partie 2 Informations détaillées sur l'étude
Partie 3 Protection des données et couverture d'assurance
Partie 4 Déclaration de consentement

Dans la **partie 1**, vous aurez un aperçu général de l'étude. Dans la **partie 2**, nous vous expliquons en détail le déroulement et le contexte de l'étude. La **partie 3** contient les informations relatives à la protection des données et à la couverture d'assurance. En signant le consentement à la fin du document, **partie 4**, vous attestez que vous avez compris toutes les informations et que vous acceptez de participer.

Cette étude est initiée par le Professeur Emmanuel Carrera, médecin adjoint agrégé, responsable de l'unité des maladies neurovasculaires du service de Neurologie des Hôpitaux Universitaires de Genève (HUG). Cette personne est appelée promoteur. Le promoteur assume la responsabilité, la gestion et le financement d'une étude.

La personne de référence pour cette étude est :

Nom	Dr. Nicolas BROC, médecin chef de clinique
Adresse	Unité des maladies neurovasculaires
	Service de Neurologie
	HUG
Téléphone	+41 79 55 33 837 (heures ouvrables)
	+41 79 55 33 960 (disponible 24h/24)
Courriel	nicolas.broc@hug.ch

Partie 1 :
L’essentiel en bref

1. Pourquoi menons-nous cette étude ?

Vous souffrez d’un AVC affectant la force et la fonction de votre bras et c'est pourquoi nous vous demandons si vous souhaitez participer à cette étude.

Chez les personnes souffrant de cette maladie, il n’existe jusqu’aujourd’hui aucun médicament qui améliore la récupération de la fonction du bras.

Dans cette étude, nous examinons comment le médicament à l’étude, le maraviroc, agit et évaluons son efficacité sur la récupération de la fonction du bras, sa tolérance, ainsi que sa sécurité.

Vous en apprendrez davantage sur le contexte scientifique de l’étude au **chapitre 4**.

2. Que devez-vous faire si vous participez ?

Votre participation durera 6 mois (3 mois de traitement et 3 mois de suivi supplémentaire). Nous vous inviterons à 6 consultations (2 dans les premiers jours de votre hospitalisation, à 1 mois, 2 mois, 3 mois et 6 mois). Lors du deuxième rendez-vous durant votre hospitalisation, ainsi qu’à celui de 3 mois, vous réaliserez une Imagerie par Résonance Magnétique (IRM). Les rendez-vous à 1, 2 et 6 mois, durent en moyenne 1h. Les rendez-vous lors de votre hospitalisation durent en moyenne 3h et l’IRM dure 30 minutes.

Les rendez-vous sont détaillés dans **le tableau au chapitre 5**.

Si vous décidez de participer, vous ferez partie soit du groupe d’intervention, soit du groupe témoin. Vous serez affecté de manière aléatoire à l’un des 2 groupes. Vous ne saurez pas à quel groupe vous appartenez. Dans le groupe d’intervention, vous recevrez le médicament à l’étude, le maraviroc. Dans le groupe témoin, vous recevrez une substance inactive, le placebo.

Vous en apprendrez plus sur le déroulement et les procédures de l’étude au **chapitre 5**.

3. Quels sont les bénéfices et les risques liés à la participation ?

Bénéfices

Vous n'allez peut-être pas obtenir de bénéfice direct en participant à cette étude. Votre participation peut toutefois contribuer à aider de futurs patients et à mieux guider leur prise en charge. Les bénéfices éventuels sont une amélioration de la récupération de la fonction de votre bras.

Risques

Le maraviroc n'est pas encore autorisé en Suisse pour améliorer la fonction motrice après AVC. Il est actuellement autorisé uniquement comme antiviral chez les patients souffrant d'une infection au virus d'immunodéficience humaine (VIH).

Des effets indésirables peuvent survenir si vous prenez le maraviroc. Bien que ce médicament soit considéré comme sûr chez les patients souffrant d'un VIH, chez qui il a déjà été testé, nous ne connaissons pas encore tous les risques et les effets indésirables. Jusqu'à présent, les risques et effets indésirables suivants sont connus :

- Diarrhées et/ou nausées.
- Maux de tête.
- Certaines infections virales (en particulier des infections virales des voies respiratoires supérieurs/rhumes)
- Perturbation des marqueurs sanguins liés au foie

Vous trouverez davantage d'informations sur les risques et les contraintes au **chapitre 6**.

Partie 2 :
Informations détaillées sur l'étude

4. Contexte scientifique

4.1 Contexte : pourquoi menons-nous cette étude ?

L'AVC est l'une des maladies les plus fréquentes et entraîne souvent des troubles neurologiques, notamment une faiblesse d'un côté du corps, qui peuvent entraîner un handicap à long terme. L'AVC est l'une des causes les plus fréquentes d'handicap dans le monde. Malgré les thérapies comme la physiothérapie ou l'ergothérapie, la récupération des troubles moteurs est souvent incomplète et le patient peut garder des séquelles à long terme.

Il n'y a, à l'heure actuelle, aucun traitement médicamenteux approuvé au-delà de la phase aiguë qui permette d'améliorer cette récupération.

Les études menées jusqu'à présent chez des animaux ont montré que le maraviroc permettrait d'améliorer la récupération des fonctions motrices et cognitives après un AVC. Nous savons également que les patients qui présentent certaines mutations génétiques identiques à l'effet du maraviroc récupèrent mieux d'un AVC.

A l'heure actuelle, il n'existe pas encore d'étude ayant directement étudié l'effet du maraviroc sur la récupération motrice chez l'homme

Nous examinons donc dans cette étude si le maraviroc est efficace sur la récupération motrice et s'il est sûr, comparé à une substance inactive (un placebo), chez les personnes venant de souffrir d'un AVC.

Le maraviroc n'est jusqu'à présent autorisé en Suisse que pour traiter certains patients infectés par le VIH. Ce n'est que lorsque l'efficacité de ce traitement aura été scientifiquement étudiée et prouvée que le maraviroc pourra être utilisé en Suisse pour améliorer la récupération motrice après un AVC.

En plus de l'effet clinique sur la motricité, nous étudions les mécanismes d'action du maraviroc, en particulier, s'il améliore la plasticité cérébrale et la survie de neurones dans la zone entourant celle endommagée par l'AVC. C'est pour cela que nous vous demandons de faire un type d'IRM cérébrale particulier, appelé une IRM fonctionnelle d'une part et une imagerie par spectroscopie magnétique d'autre part. Il s'agit de méthodes non-irradiantes, sans injection de produit de contraste, donc sans risque significatif.

4.2 Structure de l’étude : comment procédons-nous ?

Dans notre étude, les participants sont répartis au hasard dans des groupes, c’est ce qu’on appelle la randomisation. Cette méthode est importante pour obtenir des résultats fiables. Chaque groupe reçoit un traitement différent. Dans notre étude, il y a 2 groupes :

- Le **groupe 1** (groupe d’intervention) reçoit le maraviroc à une dose de 300 mg 2 fois par jour le matin et le soir, pendant une durée de 3 mois (12 semaines).
- Le **groupe** 2 (groupe de contrôle) reçoit un placebo, c’est-à-dire un comprimé sans principe actif, également 2 fois par jour le matin et le soir.

Il s’agit d’une étude dite en « double aveugle », ce qui signifie qu’aucune des personnes impliquées dans sa réalisation ne sait qui est dans quel groupe : ni les participants, ni les médecins-investigateurs ne connaissent la répartition entre les groupes. C’est pourquoi on parle de « double aveugle ». Cette méthode est choisie pour que les personnes influencent le moins possible les résultats de l’étude. La randomisation et le double aveugle nous permettent d’évaluer objectivement l’efficacité et la sécurité du médicament à l’étude.

4.3 Réglementation de la recherche scientifique impliquant des êtres humains

Nous réalisons cette étude conformément aux lois en vigueur en Suisse (loi relative à la recherche sur l’être humain, lois sur la protection des données). En outre, nous respectons toutes les directives reconnues au niveau international. La commission d’éthique compétente et Swissmedic ont examiné et autorisé l’étude.

Notre étude est une étude nationale. Il y a 80 participants.

Vous trouverez également une description de cette étude sur le site Internet de l’Office fédéral de la santé publique, à l’adresse www.kofam.ch, sous le numéro d’enregistrement SNCTP 000006328 ou le numéro BASEC 2024-02359

5. Déroulement de l'étude

5.1 Que devez-vous faire si vous participez à l'étude ?

La participation à l'étude est volontaire et dure 6 mois. Vous devez respecter le calendrier des rendez-vous (→ chapitre 5.2) ainsi que toutes les consignes données par l'équipe de recherche.

Vous devez informer l'équipe de recherche

- si votre état de santé évolue, par exemple si vous vous sentez moins bien ou si vous présentez de nouveaux troubles ; vous devez continuer de l'en informer si vous vous retirez de l'étude (→ chapitres 5.3 et 5.4) ;
- si vous débutez un nouveau médicament, complément alimentaire ou composé à base de plantes, qu'il ait été prescrit ou non par un médecin.

Vous devez également tenir compte des points suivants :

- Vous devez éviter de débuter une grossesse pendant votre participation (→ chapitre 5.5).

5.2 Que se passe-t-il lors des rendez-vous ?

Vous serez vu 6 fois au centre d'étude (les deux premiers rendez-vous ont lieu alors que vous êtes encore hospitalisé). Le deuxième rendez-vous, ainsi que celui à 3 mois, durent environ 3 heures et vous réaliserez une IRM d'environ 30 minutes. Le premier rendez-vous ainsi que les rendez-vous à 1 mois, 2 mois et 6 mois sont plus courts et durent environ 1h30. La liste des rendez-vous figure dans le tableau ci-après.

Voici ce que nous faisons à tous les rendez-vous :

- Nous répondons à vos questions.
- Nous vous posons des questions sur votre état de santé, effectuons un examen clinique et mesurons certains paramètres vitaux comme la tension artérielle, le pouls…
- Une prise de sang lors de laquelle nous prélevons 10 millilitres (ce qui correspond environ à deux cuillères à café).
- Un examen ECG, c'est-à-dire une analyse du rythme cardiaque par des électrodes posées sur votre thorax.
- Un échantillon d'urine pour effectuer un test de grossesse si vous êtes une femme en âge d'avoir des enfants.

Lors de certains rendez-vous (le premier à l’hôpital et celui à 3 mois), nous faisons en outre :

- des tests cliniques neurologiques et des échelles cliniques plus détaillées pour évaluer la récupération après votre AVC.
- une IRM, lors de laquelle on place une antenne (semblable à un casque) sur votre tête et vous couche sur une table afin de vous introduire dans un très grand aimant (semblable à un tunnel).

Ces examens nous permettent d‘évaluer l’efficacité et la sécurité du médicament à l’étude.

Plan détude:

Phases de l'étude	Pré-inclusion	Phase de traitement				Suivi
Numéro de visite	1	2	3	4	5	6
Type de RDV	présentiel	présentiel	présentiel	présentiel	présentiel	présentiel
Temps (jours)	-5 - 0	0	30 +/- 5	60 +/- 5	90 +/- 7	180 +/- 14
Mesures administratives						
Information de l’étude et déclaration de consentement	X					
Démographie et biométrie	X					
Antécédents médicaux	X					
Critères d’inclusion/d’exclusion	X					
Randomisation		X				
Procédures cliniques/évaluations						
Examen physique	X	X	X	X	X	X
Signes vitaux	X	X	X	X	X	X
Prélèvements sanguins	X	X	X	X	X	X
Test de grossesse	X	X	X	X	X	X
Electrocardiogramme	X	X	X	X	X	X
IRM fonctionnelle et spectroscopie		X			X	
Echelles et scores cliniques		X			X	
Suivi des effets indésirables		<===============en continu===============>				X
Administration du traitement de l'étude						
Durée du traitement		<===============en continu===============>				
Remise des médicaments pour 30+7 jours		X	X	X		

5.3 Quand la participation à l’étude prend-elle fin ?

Votre participation dure 6 mois et se termine après le 6ème rendez-vous. Vous pouvez interrompre votre participation à tout moment avant cette date (→ chapitre 5.4). Vous n’avez pas besoin de vous justifier. Si vous souhaitez mettre fin à votre participation, veuillez en informer le médecin-investigateur/la médecin-investigatrice.

Si vous vous retirez de l’étude, cela n'aura aucune incidence sur vos soins médicaux et votre traitement (→ chapitre 5.4 pour les autres possibilités de traitement). Dans ce cas, nous procéderons à un dernier examen, dans le cadre de l’étude, pour votre sécurité. Nous vous demanderons de nous rapporter tous les médicaments restants de l’étude.

En cas d’arrêt avant la date prévue, nous vous prions de continuer d’informer le médecin-investigateur si votre état de santé évolue, par exemple si vous vous sentez moins bien ou si de nouveaux troubles apparaissent. Si vous vous retirez de l’étude, nous pourrons encore

analyser les données et les échantillons recueillis jusque-là (p. ex. valeurs sanguines, résultats des ECG ou des IRM). Vos données et échantillons d'étude restent codés (→ chapitre 9).

Il se peut également que nous devions vous exclure de l'étude plus tôt. C'est par exemple le cas si vous ne tolérez pas le médicament à l'étude, si vous présentez des effets indésirables graves ou encore si vous devez débuter certains traitements qui interagissent avec le médicament à l'étude.

5.4 Que se passe-t-il si vous ne souhaitez pas participer ?

Si vous ne participez pas à cette étude, votre traitement et votre prise en charge médicale seront assurés conformément aux standards actuels. Si vous ne souhaitez pas participer à l'étude, cela n'aura aucune incidence sur vos soins médicaux et votre traitement

5.5 Grossesse

Le médicament à l'étude n'est pas autorisé pendant la grossesse ou l'allaitement. Il pourrait être dangereux et nocif pour un enfant à naître ou qui allaite.

Vous ne pouvez donc pas avoir d'enfants pendant votre participation à l'étude. Cela vaut pour les femmes et les hommes qui participent à cette étude. Vous discuterez de ces questions avec le médecin-investigateur.

Pour les femmes qui peuvent débuter une grossesse

Vous devez éviter de débuter une grossesse pendant votre participation à l'étude. Vous devez informer votre partenaire / vos partenaires que vous prenez part à cette étude. Avant de commencer, vous ferez un test de grossesse urinaire. Des tests de grossesse (urinaires) seront ensuite effectués régulièrement. Si vous allaitez, vous ne pouvez pas participer à l'étude.

Pendant votre participation à l'étude, vous devez utiliser une méthode de contraception efficace.

1. une préparation qui supprime l'ovulation, soit sous forme de comprimé (« pilule »), d'injection, de bâtonnet sous la peau, de patch ou d'anneau vaginal ou
2. un stérilet hormonal ou un stérilet au cuivre.

Vous devez continuer d'utiliser ces méthodes de contraception pendant au moins 3 mois après la dernière dose du traitement à l'étude. Si vous débutiez malgré tout une grossesse au cours de l'étude ou jusqu'à 3 mois après la dernière dose du traitement à l'étude, vous devez en informer immédiatement le médecin-investigateur/la médecin-investigatrice. Il/Elle discutera avec vous et votre partenaire de la suite à donner.

6. Risques, contraintes et effets indésirables

6.1 Quels sont les risques et les contraintes liés à l'étude ?

La participation à cette étude comporte des risques et des contraintes, comme tout traitement médical. Nous connaissons déjà certains risques, d'autres sont encore inconnus. Cette incertitude n'est pas inhabituelle dans un contexte d'étude. Vous trouverez au **chapitre 6.2** une liste des risques les plus fréquents et les plus graves. De nombreux effets indésirables peuvent être traités médicalement. Nous vous informerons pendant l'étude de toutes nouvelles connaissances sur les risques et les effets indésirables.

Le médicament à l'étude a déjà été utilisé par de nombreuses personnes souffrant d'une infection au VIH, avec un profil favorable d'effet indésirables. Cependant, les caractéristiques des sujets dans les études pour le traitement contre le VIH ne sont pas toujours identiques à celles des patients de notre étude et il se peut que certains effets indésirables soient plus/moins fréquents de ce qui est jusqu'alors connu.

Les examens médicaux que nous effectuons dans le cadre de l'étude comportent également des risques. Certains examens font d'ores et déjà partie de votre prise en charge standard et votre médecin vous en a présenté les risques. Vous trouverez au **chapitre 6.3** une liste des risques liés aux examens.

6.2 Risques les plus fréquents et les plus graves liés au médicament à l'étude

Vous trouverez ici des informations sur les effets indésirables les plus fréquents et les plus graves que nous connaissons déjà.

Nous utilisons les catégories suivantes pour les décrire :

Très fréquent	L'effet indésirable apparaît chez plus de 10 personnes sur 100 (plus de 10 %).
Fréquent	L'effet indésirable apparaît chez 1 à 10 personnes sur 100 (1 %-10 %).
Occasionnel	L'effet indésirable apparaît chez 1 à 10 personnes sur 1000 (0,1%-1%).
Rare	L'effet indésirable apparaît chez 1 à 10 personnes sur 10 000 (0,01 %-0,1 %).
Très rare	L'effet indésirable apparaît chez moins d'une personne sur 10 000 (moins de 0,01 %).

Les effets indésirables très fréquents et fréquents connus sont (du plus fréquent au moins fréquent) :

	**Maraviroc 2 fois par jour N=246 (%)**	**Placebo 2 fois par jour N=209 (%)**
Très fréquent (> 10 %)		
Infections des voies respiratoires supérieures	22.8	12.9
Toux et symptômes associés	13.8	5.3
Fièvre	12.9	8.6
Eruption cutanée	10.8	5.3
Fréquent (1 - 10 %)		
Sensation vertigineuse/vertige postural	8.7	8.1
Infection herpétique	7.7	4.3
Troubles de l'endormissement et du maintien du sommeil	7.7	5.3
Troubles de l'appétit	7.5	6.7
Sinusite	6.8	3.3
Signes et symptômes liés aux articulations	6.8	2.9
Bronchite	6.6	4.8
Signes et symptômes liés aux voies respiratoires supérieures	6.1	3.3
Constipation	5.9	2.9
Paresthésies et dysesthésies	4.9	2.9

Symptômes de la vessie et de l'urètre	4.9	1.4
Troubles des glandes eccrines et apocrines	4.9	3.8
Maladies du derme et de l'épisderme	4.5	4.3
Troubles dépressifs	4.2	2.9
Congestion et inflammation nasales	4.2	2.9
Troubles sensoriels	4	1.4
Douleurs et inconfort	3.8	2.9
Folliculite	3.8	1.9
Troubles de la conscience	3.8	2.9
Neuropathies périphériques	3.8	2.9
Prurit	3.8	1.9
Troubles anxieux	3.5	3.3
Troubles de la respiration	3.5	2.4
Oedème	3.3	2.9
Lipodystrophies	3.3	0.5
Signes et symptômes généraux	3.1	2.4
Douleurs musculaires	3.1	0.5
Néoplasies bénignes de la peau	3.1	1.4
Troubles vasculaires hypertensifs	3.1	1.9
Signes et symptômes des voies urinaires	2.8	1.4
Troubles des sinus paranasaux	2.8	0.5
Infections , irritations et inflammations des conjonctives	2.3	1.4
Stomatite, ulcérations	2.3	1.9
Signes et symptômes gastro-intestinaux	2.3	1.9
Pneumonie	2.3	5.3
Erythème	2.3	1
Infections et inflammations oculaires et manigfestations associées	2.1	1
Perception de la température corporelle	2.1	1.9
Verrues anogénitales	2.1	1.4
Grippe	2.1	0.5
Otite moyenne	2.1	0.5
Bronchospasme et obstruction	2.1	1.9

En particulier, les effets indésirables fréquents plus graves sont :

- Perturbation des tests hépatiques (5.16%)
- Fièvre (2.58%)

Les effets indésirables rares et très rares sont :

- Éruption cutanée généralisée.
- Inflammation des muscles (myosite)
- Nausées et vomissements importants.
- Perte de connaissance.
- Diminution des cellules sanguines (pancytopénie).
- Pneumonie.
- Cancer de l'œsophage, des voies biliaires, métastases au foie, os et péritoine (mais moins de cas que dans le groupe placebo).
- Insuffisance du foie (avec rares cas de caractéristiques allergiques).

6.3 Risques et contraintes liés aux examens dans le cadre de l'étude

Nous effectuons différents examens médicaux pour cette étude (→ chapitre 5.2). Ces examens sont des procédures éprouvées. Néanmoins, ils peuvent comporter des risques et des contraintes, c'est-à-dire qu'ils peuvent être désagréables ou avoir des effets indésirables. Dans cette étude, les risques et les contraintes sont les suivants :

- Prise de sang : des hématomes, des saignements ou des gonflements peuvent survenir au point de ponction.
- Électrocardiogramme (ECG) : l'ECG est un enregistrement de l'activité électrique du cœur. On utilise des électrodes adhésives qui peuvent provoquer une réaction de la peau.
- IRM : lors d'une IRM la personne est placée dans un tube avec une antenne sur la tête (semblable à un casque). Cela peut engendrer une claustrophobie chez les individus susceptibles. Il n'y a pas d'injection de produit de contraste ni d'irradiation.

7. Financement et indemnisation

Cette étude est initiée et financée par le promoteur-investigateur (Dr. Emmanuel Carrera) et financée par le fonds national suisse pour la recherche (FNS).

Les chercheurs participant à l'étude n'en retirent aucun avantage financier direct.

Si vous participez à cette étude, vous ne recevrez pas d'argent ni aucune autre compensation.

La participation à l'étude n'entraîne aucun coût supplémentaire pour vous ni pour votre assurance-maladie. Nous vous rembourserons les frais de déplacement occasionnés par votre participation.

Les résultats de cette étude peuvent contribuer à la commercialisation d'un médicament. Votre participation ne vous donne aucun droit concernant son exploitation commerciale.

8. Résultats de l'étude

Les résultats qui vous concernent personnellement vous sont communiqués par le médecin-investigateur/la médecin-investigatrice. Parfois, des résultats sont découverts par hasard. Il peut s'agir par exemple de résultats de la prise de sang ou de l'imagerie cérébrale. Nous vous informons si ces découvertes sont importantes pour votre santé.

Nous vous informons par exemple si nous découvrons par hasard une maladie dont vous n'avez pas encore connaissance et que nous pouvons traiter. Si vous *ne souhaitez pas* recevoir ces informations, veuillez en parler au médecin-investigateur/à la médecin-investigatrice.

En plus des résultats individuels, l'étude produira des résultats globaux qui proviennent des données de toutes les personnes y participant. Il s'agirait p.ex. de nouvelles connaissances sur les mécanismes de la récupération après un AVC (→ chapitre 4.1). Ces résultats ne vous concernent pas directement, ni votre santé. Si vous le souhaitez, le médecin-investigateur/la médecin-investigatrice vous fournira un résumé des résultats globaux à la fin de l'étude.

Partie 3 :
Protection des données et couverture d’assurance

9. Protection des données et des échantillons

Nous protégeons vos données (p. ex. les données de votre dossier médical telles que la tension artérielle et le pouls, les imageries cérébrales) et vos échantillons (p. ex. vos échantillons de sang). Les lois suisses prévoient des règles strictes en matière de protection des données et des échantillons.

La législation suisse sur la protection des données vous donne le droit d'accéder, de rectifier et de recevoir les données qui ont été collectées, traitées et transmises dans le cadre de l'étude. Dans des cas exceptionnels en raison d'autres exigences légales ou réglementaires, ces droits ne peuvent pas toujours être garantis. Si vous avez des questions à ce sujet, veuillez contacter votre médecin-investigateur.

9.1 Codage des données et des échantillons

Toute étude génère des données issues des examens (p. ex. valeurs sanguines, résultats des ECG, des IRM). Ces données sont enregistrées de manière codée, généralement sous forme électronique. Le codage signifie que les informations personnelles qui peuvent vous identifier directement sont conservées *séparément* des autres données, sous la forme d’une liste (liste d’identification) qui identifie chaque personne avec un code unique. Ainsi, votre nom, votre date de naissance ou votre adresse *ne figurent pas* directement avec les autres données collectées. Cette liste d’identification reste pendant 20 années à l’hôpital et est ensuite détruite. Personne d’autre ne la reçoit. Les exceptions particulières sont réglées au chapitre 9.5.

Lorsque nous transmettons des données dans le cadre de cette étude – au promoteur ou à des spécialistes ou organisations qui effectuent d’autres analyses –, elles sont toujours codées et vos données personnelles sont protégées.

Tous les échantillons sont également codés de cette manière. Vos données personnelles sont donc protégées lorsque nous envoyons des échantillons pour qu’ils soient analysés en laboratoire. Les données et les échantillons sont toujours codés au laboratoire.

9.2 Sécurité des données et des échantillons pendant l’étude

Le promoteur-investigateur (Dr. Emmanuel Carrera) est responsable de la sécurité de vos données et des échantillons de cette étude. Il veille au respect des lois en vigueur, par exemple des lois sur la protection des données. Cette règle s’applique également lorsque des données ou des échantillons (codés) sont envoyés pour analyse dans des pays où les lois sur la protection des données sont moins strictes. Voici comment le promoteur de l’étude protège vos données :

Dans cette étude, vos données sont saisies et transmises par voie électronique. Les données sont stockées sur un serveur en Suisse. Néanmoins, le risque que des personnes non autorisées accèdent à vos données personnelles ne peut être entièrement exclu (p. ex. risque de « piratage »).

Il peut être important que votre médecin traitant partage des données de votre dossier médical avec l’équipe de recherche. Cela vaut également pour les autres médecins qui vous suivent. En signant le consentement à la fin du document, vous autorisez la communication de ces données.

9.3 Sécurité des données et des échantillons après la fin de l’étude

Lorsque l’étude est terminée, le promoteur continue d’assurer la sécurité de vos données et échantillons. La loi prescrit que tous les documents de l’étude, par exemple les formulaires de collecte de données, doivent être conservés pendant au moins 20 ans.

Au terme de cette longue période, les données de l'étude restent codées. Les données relatives à la santé de votre dossier médical, y compris celles de cette étude, sont et resteront toujours accessibles à vos soignants.

Dans le cas où des résidus d’échantillons à la fin de l’étude, nous les collectons et les conservons sous forme codée pendant au moins 10 ans dans un endroit sûr. Ils pourront éventuellement être utilisés plus tard pour d’autres analyses (→ chapitre 9.4). Une telle collection d’échantillons codés est appelée une « biobanque ». Il existe des règles strictes pour les biobanques, afin que les informations contenues dans vos échantillons soient bien protégées.

Une fois l’étude terminée, les résultats sont généralement publiés dans des revues scientifiques. Pour ce faire, les données sont envoyées sous forme codée à d’autres spécialistes afin qu’ils puissent réviser la publication. Ces données ne peuvent pas être réutilisées à des fins de recherche. Une telle réutilisation à d’autres fins de recherche nécessite votre consentement séparé (→ chapitre 9.4).

9.4 Réutilisation et transfert de vos données et échantillons pour d’autres études

Les données et les échantillons issus de cette étude sont très importants pour la recherche future. Les échantillons qui n'ont pas encore été utilisés pour cette étude, ainsi que les

données utilisées pour cette étude, peuvent être réutilisés et/ou transmis pour d'autres études (aussi à l'étranger).

Un consentement séparé pour la réutilisation et/ou transfert de vos données génétiques et de vos échantillons est nécessaire. Celui-ci est facultatif. Nous vous invitons à lire attentivement la déclaration de consentement supplémentaire à la fin du document. Veuillez signer le consentement si vous acceptez de mettre à disposition vos données et vos échantillons pour de futures recherches. Vous pouvez participer à l'étude même si vous ne signez pas ce document additionnel.

9.5 Droit de consultation lors des contrôles

La réalisation de cette étude peut faire l'objet de contrôles. Ces contrôles sont effectués par des autorités telles que la commission d'éthique compétente ou l'autorité d'autorisation *Swissmedic*, ou encore par des autorités d'autorisation étrangères. Le promoteur doit également procéder à des vérifications afin de garantir la qualité de l'étude et de ses résultats.

Pour ces contrôles, un petit nombre de personnes spécialement formées ont accès à vos données personnelles et à votre dossier médical. Dans ce cadre, les données ne sont donc *pas* codées. Les personnes qui consultent vos données non codées sont soumises au secret professionnel.

En tant que participant·e, vous avez à tout moment le droit de consulter vos données.

10. Couverture d’assurance

Vous bénéficiez d’une couverture d’assurance si vous subissez un dommage du fait de l’étude – c’est-à-dire du fait du médicament à l’étude. La procédure est réglée par la loi. Le promoteur a conclu une assurance auprès de Baloise Assurance SA (N° police : 30/4.085.800/ 2024_02359). Si vous pensez avoir subi un dommage du fait de l’étude, veuillez-vous adresser au médecin-investigateur ou directement à l’assurance.

Si un dommage résulte de l’utilisation conforme d’un médicament autorisé ou de l’application d’un traitement conventionnel, les règles en matière de responsabilité sont les mêmes que pour les traitements en dehors d’une étude. En pareil cas, l’assurance responsabilité civile de l’hôpital prend en charge les frais / l’indemnisation.

Partie 4 : Analyse génétique: mutation CCR5-Delta-32

Dans le cadre de cette étude, une analyse génétique spécifique visant à détecter la mutation CCR5-Delta-32 sera effectuée à partir du prélèvement sanguin du patient/de la patiente sous votre responsabilité. Cette mutation génétique relativement fréquente dans la population générale est étudiée car elle pourrait influencer la récupération motrice après un accident vasculaire cérébral (AVC). Les informations obtenues permettront de mieux comprendre l'impact potentiel de cette mutation sur l'efficacité du traitement à l'étude (maraviroc).

1. Pourquoi cette analyse génétique est-elle effectuée ?

Cette analyse vise à explorer si les individus porteurs de la mutation CCR5-Delta-32 bénéficient d'une récupération motrice différente par rapport aux non-porteurs et s'ils répondent mieux, ou moins bien, au traitement de maraviroc. Ces résultats contribueront à la compréhension des mécanismes de récupération après un AVC et pourraient orienter les traitements futurs.

2. Quels sont les résultats possibles et leurs implications ?

- **Présence de la mutation** : Cela n'entraîne pas de conséquences médicales directes pour la santé du patient/de la patiente sous votre responsabilité en dehors de cette étude.
- **Absence de la mutation** : Cela signifie que cette variation génétique n'est pas présente dans l'ADN du patient/de la patiente sous votre responsabilité.
- Ces résultats n'auront aucun impact direct sur la prise en charge médicale actuelle ou future du patient/de la patiente sous votre responsabilité.

3. Protection des données et consentement

Les données génétiques du patient/de la patiente sous votre responsabilité seront strictement codées pour protéger sa confidentialité. Les résultats de cette analyse ne seront pas partagés en dehors du cadre de cette étude, sauf si vous en donnez un consentement explicite. Le patient/la patiente sous votre responsabilité peut choisir de participer à l'étude sans accepter cette analyse génétique.

Partie 5 :
Déclarations de consentement

La présente déclaration de consentement se compose de trois parties séparées :

- Déclaration de consentement pour la participation à l'étude MASTER
- Déclaration de consentement pour l'analyse génétique CCR5-Delta-32
- Déclaration de consentement pour la réutilisation et le transfert sous forme codée des données et des échantillons de cette étude pour des recherches ultérieures

Veuillez lire attentivement ce formulaire. N'hésitez pas à nous poser des questions si vous ne comprenez pas quelque chose ou si vous souhaitez des précisions. Votre consentement écrit est nécessaire pour participer.

Déclaration de consentement pour la participation à l'étude MASTER

**Numéro BASEC**	2024-02359
**Titre de l'étude**	Étude de phase II sur l'efficacité sur la récupération motrice et la sécurité du maraviroc par rapport au placebo après un accident vasculaire cérébral
**Titre simplifié**	MASTER
**Institution responsable** (promoteur et adresse)	Pr. Emmanuel Carrera<br>Médecin Adjoint Agrégé<br>Responsable de l'unité des maladies neurovasculaires<br>Service de Neurologie<br>Hôpitaux Universitaires de Genève<br>Rue Gabrielle Perret Gentil 4<br>1205 Genève
**Lieu de réalisation**	Genève, Suisse
**Médecin-investigateur responsable sur le site**	Pr. Emmanuel Carrera (promoteur-investigateur)

Participant
Nom et prénom en caractères d’imprimerie :
Date de naissance :

- J’ai reçu des informations orales et écrites sur l’étude de la part du médecin-investigateur/ de la médecin-investigatrice soussigné.e.
- Le médecin-investigateur m’a expliqué le but, le déroulement et les risques de l’étude.
- Je participe volontairement à l’étude.
- Le médecin-investigateur m’a expliqué quels étaient les traitements standards possibles en dehors de l’étude.
- J’ai eu suffisamment de temps pour prendre ma décision. Je conserve les informations écrites et je reçois une copie de ma déclaration de consentement écrite.
- Je peux mettre fin à ma participation à tout moment, je n’ai pas besoin de me justifier. Même si je me retire de l’étude, ma prise en charge médicale reste assurée. Les données et les échantillons collectés jusque-là restent enregistrés et seront encore analysés dans le cadre de l’étude.
- Le médecin-investigateur peut m’exclure de l’étude à tout moment dans l’intérêt de ma santé.
- Les résultats et/ou les découvertes fortuites qui concernent directement ma santé me seront communiqués. Si je ne le souhaite pas, j’en informe le médecin-investigateur.
- Mon médecin traitant doit être informé de ma participation à l’étude. Il peut partager avec le médecin-investigateur des données de mon dossier médical qui sont importantes pour l’étude. Cela vaut également pour les autres médecins qui me suivent.
- Les spécialistes compétents du promoteur, de la commission d’éthique et de l’autorité de contrôle des médicaments *Swissmedic* peuvent consulter mes données non codées à des fins de contrôle. Toutes ces personnes sont soumises au secret professionnel.
- Je sais que les HUG ont souscrit une assurance. Cette assurance paie si je subis un dommage – mais seulement si le dommage est directement lié à l’étude.

Lieu, date	Nom et prénom du participant en caractères d’imprimerie<br><br><br><br>Signature du participant

Attestation du médecin-investigateur : Par la présente, j’atteste avoir expliqué au participant la nature, l’importance et la portée de l’étude. Je déclare satisfaire à toutes les obligations en relation avec cette étude selon le droit suisse. Si je devais prendre connaissance, au cours de l’étude, d’éléments susceptibles d’influer sur la disposition du participant à prendre part à l’étude, je m’engage à l’en informer immédiatement.

Lieu, date	Nom et prénom du médecin-investigateur en caractères d’imprimerie<br><br><br><br>Signature du médecin-investigateur

Déclaration de consentement écrite pour l'analyse génétique CCR5-Delta-32

Veuillez lire attentivement ce formulaire. N'hésitez pas à nous poser des questions si vous ne comprenez pas quelque chose ou si vous souhaitez des précisions. Votre consentement écrit est nécessaire pour participer.

**Numéro BASEC du projet de recherche (après soumission à la commission d'éthique compétente) :**	2024-02359
**Titre (scientifique et usuel) :**	Étude de phase II sur l'efficacité sur la récupération motrice et la sécurité du maraviroc par rapport au placebo après un accident vasculaire cérébral
**Institution responsable (promoteur et adresse complète) :**	Pr. Emmanuel Carrera<br>Médecin Adjoint Agrégé<br>Responsable de l'unité des maladies neurovasculaires<br>Service de Neurologie<br>Hôpitaux Universitaires de Genève<br>Rue Gabrielle Perret Gentil 4<br>1205 Genève
**Lieu de réalisation :**	Genève, Suisse
**Médecin-investigatrice/investigateur responsable sur le site :**<br>Nom et prénom en caractères d'imprimerie :	Pr. Emmanuel Carrera (promoteur-investigateur)
**Participant·e :**<br>Nom et prénom en caractères d'imprimerie :<br>Date de naissance :	

J'ai reçu des informations orales et écrites de la part du médecin-investigateur / de la médecin-investigatrice soussigné·e sur l'objectif et le déroulement de l'analyse génétique prévue dans cette étude, à savoir l'analyse de la mutation CCR5-Delta-32. Cette analyse vise à explorer son rôle potentiel dans la récupération motrice après un AVC, ainsi que ses avantages et risques éventuels.

Le médecin-investigateur m'a expliqué le but, le déroulement et les risques de cette analyse génétique, ainsi que les implications possibles des résultats.

- Je participe volontairement à cette analyse génétique dans le cadre de l'étude clinique.
- Le médecin-investigateur m'a informé des traitements standards possibles en dehors de cette étude et m'a expliqué que cette analyse génétique n'aura pas de conséquence directe sur ma prise en charge médicale actuelle.
- J'ai eu suffisamment de temps pour prendre ma décision. Je conserve les informations écrites et je reçois une copie de ma déclaration de consentement écrite.
- Je peux mettre fin à ma participation à cette analyse génétique à tout moment et sans avoir à me justifier. Cette décision n'aura aucune répercussion sur ma prise en charge médicale ou sur ma participation à l'étude principale. Les données recueillies jusqu'à ce moment resteront enregistrées et pourront être analysées dans le cadre de l'étude, conformément à la loi.
- Les résultats de cette analyse me seront communiqués s'ils concernent directement ma santé. Si je ne souhaite pas recevoir ces résultats, j'en informe le médecin-investigateur.
- Mon médecin traitant sera informé de ma participation à cette analyse génétique et pourra, si nécessaire, partager avec le médecin-investigateur des données importantes pour l'étude.
- Les spécialistes compétents du promoteur, de la commission d'éthique et de Swissmedic peuvent consulter mes données non codées pour des contrôles et des inspections, dans le respect de la confidentialité. Toutes ces personnes sont soumises au secret professionnel.
- Je suis conscient·e que les données génétiques recueillies seront codées pour garantir leur confidentialité et ne seront utilisées que dans le cadre de cette étude.

Lieu, date	Nom et prénom du participant en caractères d'imprimerie<br><br><br>Signature du participant

Attestation de la médecin-investigatrice/du médecin-investigateur : Par la présente, j'atteste avoir expliqué au participant l'importance et la portée de l'analyse génétique. Je déclare satisfaire à toutes les obligations en relation avec cette étude conformément au droit suisse en vigueur. Si je devais, à quelque moment que ce soit durant la réalisation de l'étude, prendre connaissance d'éléments susceptibles d'influer sur le consentement de la paticipante/du participant, je m'engage à en informer immédiatement la personne.

Lieu, date	Nom et prénom de la médecin-investigatrice/du médecin-investigateur en caractères d’imprimerie.<br><br><br>Signature de la médecin-investigatrice/du médecin-investigateur

Déclaration de consentement pour la réutilisation et/ou transfert de données (génétiques) et d'échantillons sous forme codée

Ce consentement ne porte pas sur votre participation individuelle à une étude (→ chapitre 9.4 de la feuille d'information).

La « réutilisation » signifie que vos données et vos échantillons peuvent être conservés au-delà de votre participation à l'étude et utilisés sous forme codée pour d'autres études. Par exemple, un échantillon de sang et les valeurs de laboratoire vous concernant peuvent être analysés avec un grand nombre d'autres données, ou de nouvelles analyses peuvent être effectuées avec ces données.

« Transfert » signifie que vos données et échantillons peuvent être transmis à d'autres chercheurs ou institutions de recherche sous une forme codée pour d'autres études. Ces autres chercheurs ou institutions de recherche peuvent se trouver à l'étranger. Il est de la responsabilité du promoteur de s'assurer que ce pays dispose d'un niveau de protection des données adéquat, comparable à celui de la Suisse.

**Numéro BASEC**	2024-02359
**Titre de l'étude**	Étude de phase II sur l'efficacité sur la récupération motrice et la sécurité du maraviroc par rapport au placebo après un accident vasculaire cérébral
**Titre simplifié**	MASTER
**Participant**<br>Nom et prénom en caractères d'imprimerie :<br>Date de naissance :	

- J'autorise la réutilisation et le transfert sous forme codée de mes données et des échantillons de cette étude à des fins de recherche médicale (aussi à l'étranger). Les échantillons seront stockés dans une biobanque. Ils seront ensuite disponibles pour de futurs projets de recherche, pour une durée indéterminée.

- J'ai compris que les données et les échantillons sont codés et que la liste d'identification est conservée en toute sécurité.

- Les données peuvent être analysées en Suisse et à l'étranger et stockées dans une banque de données en Suisse ou à l'étranger. Les échantillons peuvent être analysés en Suisse ou à l'étranger et stockés dans une biobanque. Les institutions de recherche à l'étranger doivent respecter les mêmes normes de protection des données que celles en vigueur en Suisse.

- Je prends ma décision de mon plein gré de la réutilisation et/ou du transfert de données et d'échantillons sous forme codée et je peux revenir sur ma décision à tout moment. J'informe simplement le médecin-investigateur/la médecin-investigatrice de ma décision. Je n'ai pas à me justifier.

- Si je décide de mettre fin à ma participation, les données resteront codées et les échantillons seront détruits.

- En principe, les données et les échantillons sont analysés de manière globale. Si, par hasard, un résultat très important pour ma santé apparaît, la personne responsable prendra contact avec moi. Si je ne le souhaite pas, j'en parle avec le médecin-investigateur/la médecin-investigatrice.

Lieu, date	Nom et prénom du participant en caractères d'imprimerie<br><br><br>Signature du participant

Attestation du médecin-investigateur : J'atteste avoir expliqué au participant la nature, l'importance et la portée de la réutilisation et/ou transfert des échantillons et/ou des données génétiques.

Lieu, date	Nom et prénom du médecin-investigateur en caractères d'imprimerie<br><br><br>Signature du médecin-investigateur
