# Supplementary material for: Maraviroc for Stroke Recovery (MASTER): protocol for a phase 2 double-blind placebo-controlled randomised clinical trial
Source: BMJ Open. 2026 May 3;16(4):e109554. doi: 10.1136/bmjopen-2025-109554 (PMC13141164; doi:10.1136/bmjopen-2025-109554)
Supplement: Supplementary file 2 [file bmjopen-2025-109554.reviewer_comments.pdf]

PEER REVIEW HISTORY

BMJ Open publishes all reviews undertaken for accepted manuscripts. Reviewers are asked to complete a checklist review form and are provided with free text boxes to elaborate on their assessment. These free text comments are reproduced below.

ARTICLE DETAILS

Title (Provisional)

Maraviroc for Stroke Recovery (MASTER): protocol for a phase 2 double-blind placebo controlled randomized clinical trial

Authors

Broc, Nicolas; Byczynski, Gabriel; Dirren, Elisabeth; Carrera, Emmanuel

---

VERSION 1 - REVIEW

**Reviewer**	**1**
**Name**	**Osanai, Toshiya**
**Affiliation**	**Hokkaido Daigaku Byoin, Neurosurgery**
**Date**	**08-Jan-2026**
**COI**	**None**

The manuscript presents a protocol for the MASTER (MAraviroc for STrokE Recovery) trial, a phase 2, single-center, randomized, double-blind, placebo-controlled clinical trial. The study investigates the efficacy and safety of Maraviroc, a CCR5 antagonist, in improving motor recovery of the upper limb following ischemic stroke.

The trial’s design is highly innovative, particularly in its multimodal approach that combines clinical motor assessments with high-resolution MRI connectivity and spectroscopy to explore underlying neuroplastic mechanisms. Furthermore, the plan to initiate treatment within 7 days of stroke onset is a significant strength, as it targets the critical early subacute window of plasticity. While the protocol is generally well-structured and follows the SPIRIT 2025 guidelines, several methodological details require further clarification to ensure the robustness of the findings.

1. The protocol explicitly states that all patients will receive standard neurorehabilitation in accordance with European guidelines. Furthermore, the statistical analysis plan includes using "weekly rehabilitation hours" as a covariate. However, given that the efficacy of pharmacological interventions often depends on a synergistic effect with physical therapy, there is a concern that the "quality" and "specific content" of the rehabilitation (e.g., task-oriented training) could act as confounding factors for the results. Therefore, to ensure

consistency between the intervention and placebo groups, it is recommended that minimum standards or specific implementation criteria for the rehabilitation program be defined.

2. This study includes a plan to perform genotyping for the CCR5-Δ32 mutation. The introduction acknowledges that this loss-of-function mutation is associated with improved spontaneous recovery in stroke patients4. Since maraviroc is a drug that targets the CCR5 receptor, the inclusion of patients who already carry this mutation may mask the drug's efficacy. Please clarify whether these participants will be excluded or if a stratified analysis based on genotype will be performed to account for potential baseline bias.

3. The sample size calculation (n = 80) utilizes a pooled standard deviation (SD) of 17.5 cited from the FLAME trial. However, the FLAME trial investigated fluoxetine, which has a different mechanism of action than maraviroc. Please provide a brief justification for why the SD derived from a trial of a selective serotonin reuptake inhibitor is considered an appropriate proxy for a trial involving a CCR5 antagonist.

VERSION 1 - AUTHOR RESPONSE

Reviewer comments:

1. The manuscript presents a protocol for the MASTER (MAraviroc for STrokE Recovery) trial, a phase 2, single-center, randomized, double-blind, placebo-controlled clinical trial. The study investigates the efficacy and safety of Maraviroc, a CCR5 antagonist, in improving motor recovery of the upper limb following ischemic stroke.
   The trial’s design is highly innovative, particularly in its multimodal approach that combines clinical motor assessments with high-resolution MRI connectivity and spectroscopy to explore underlying neuroplastic mechanisms. Furthermore, the plan to initiate treatment within 7 days of stroke onset is a significant strength, as it targets the critical early subacute window of plasticity. While the protocol is generally well-structured and follows the SPIRIT 2025 guidelines, several methodological details require further clarification to ensure the robustness of the findings.

We thank the reviewer for their thorough and thoughtful evaluation of our protocol and for the positive and constructive comments. We appreciate the recognition of the innovative aspects of the trial design and the strengths of the multimodal and early-intervention approach. We have carefully addressed the points raised below and provided clarifications to strengthen the methodological aspects of the protocol.

2. The protocol explicitly states that all patients will receive standard neurorehabilitation in accordance with European guidelines. Furthermore, the statistical analysis plan includes using "weekly rehabilitation hours" as a

covariate. However, given that the efficacy of pharmacological interventions often depends on a synergistic effect with physical therapy, there is a concern that the "quality" and "specific content" of the rehabilitation (e.g., task-oriented training) could act as confounding factors for the results. Therefore, to ensure consistency between the intervention and placebo groups, it is recommended that minimum standards or specific implementation criteria for the rehabilitation program be defined.

We thank the reviewer for raising this important point. We agree that variability in rehabilitation content could act as a potential confounder, particularly given the hypothesised interaction between pharmacological treatment and neuroplasticity.

Detailed information on the content and focus of *inpatient* rehabilitation sessions will be available and recorded, and inpatient therapy follows the institutional stroke rehabilitation programme in accordance with European Stroke Organisation guidelines[1], which is currently recommended to include a minimum of 20 hours of intensive repetitive arm training 3–5 times per week over 4–6 weeks. For *outpatient* rehabilitation, the exact content of sessions cannot be recorded and is therefore acknowledged as a limitation of the study. Rehabilitation exposure in the outpatient setting will be quantified by weekly therapy hours. This will enable controlling both for the number of hours of therapy received and percentage of patients meeting ESO guideline requirements.

The section of the manuscript reading: “Intensive neurorehabilitation (…) or outpatient setting”, has been modified to : “All participants will receive standard neurorehabilitation according to our institutional stroke rehabilitation programme, aligned with European Stroke Organisation guidelines [9], which is currently recommended to include a minimum of 20 hours of intensive repetitive arm training 3–5 times per week over 4–6 weeks. During inpatient rehabilitation, detailed information on therapy content and focus (including upper-limb–specific and task-oriented training) will be recorded. During outpatient rehabilitation, rehabilitation exposure will be quantified by weekly therapy hours. This will enable controlling both for the number of hours of therapy received and percentage of patients meeting ESO guideline requirements, based on the reported therapy content.”

3. This study includes a plan to perform genotyping for the CCR5-Δ32 mutation. The introduction acknowledges that this loss-of-function mutation is associated with improved spontaneous recovery in stroke patients4. Since maraviroc is a drug that targets the CCR5 receptor, the inclusion of patients who already carry this mutation may mask the drug's efficacy. Please clarify whether these participants will be excluded or if a stratified analysis based on genotype will be performed to account for potential baseline bias.

We thank the reviewer for this insightful comment. Participants carrying the CCR5-Δ32 loss-of-function mutation will not be excluded from the trial. However, to account for the potential influence of genotype on motor recovery and the efficacy of maraviroc, a stratified analysis based on CCR5 genotype will be performed. This approach has been clarified in the "**Statistical methods for primary and secondary outcomes**" section of the revised manuscript (added sentence highlighted hereafter):

An adjusted analysis using multivariate linear regression will account for stratification variables (infarct side, finger extension ability) as well as other variables like CCR5-Δ32 loss-of-function mutation carrier status to account for confounding effects on motor recovery and treatment response.

4. The sample size calculation (n = 80) utilizes a pooled standard deviation (SD) of 17.5 cited from the FLAME trial. However, the FLAME trial investigated fluoxetine, which has a different mechanism of action than maraviroc. Please provide a brief justification for why the SD derived from a trial of a selective serotonin reuptake inhibitor is considered an appropriate proxy for a trial involving a CCR5 antagonist.

We thank the reviewer for this important point. We have based our calculation of sample size on the standard deviation of the FLAME trial because, like MASTER, it enrolled patients with moderately severe motor deficits in the acute-early subacute phase after ischemic stroke. We used the results of the FLAME study because it contains the best available estimate for variability in upper-limb function in a pharmacological RCT studying recovery in a comparable patient population. No prior study has investigated the efficacy of Maraviroc on motor recovery.

We have now included the following sentence in the section "Sample size estimates":

We used the results of the FLAME RCT, which investigated the effect of fluoxetine on stroke recovery. We based our estimation on this study because it is a large pharmacological RCT focusing on a similar population including patients with moderately severe motor deficits early after ischemic stroke. No studies investing maraviroc after stroke currently exists.

We hope that these revisions and clarifications adequately address the reviewers' and editor's comments, and we thank them again for their constructive and insightful feedback, which has helped us improve the clarity and rigor of the protocol.

<u>References</u>

1. Alt Murphy M, Munoz-Novoa M, Heremans C, Branscheidt M, Cabanas-Valdés R, Engelter ST, Kruuse C, Kwakkel G, Lakičević S, Lampropoulou S, et al. European Stroke Organisation (ESO) guideline on motor rehabilitation. *European Stroke Journal*. 2025;10:1160-1188. doi: 10.1177/23969873251338142

VERSION 2 - REVIEW

**Reviewer**	**1**
**Name**	**Osanai, Toshiya**
**Affiliation**	**Hokkaido Daigaku Byoin, Neurosurgery**
**Date**	**15-Mar-2026**
**COI**	

We are deeply grateful to the authors for their thorough and conscientious responses to all of the comments raised. Having carefully considered the revisions and clarifications provided, we are satisfied that all concerns identified in the course of this review have been adequately addressed. The protocol represents a methodologically rigorous and clinically innovative study design of a high standard. We would like to express our sincere appreciation to the authors for their diligent and thoughtful engagement with the review process.
